# Stressful urban walks: an experimental design for measuring physiological and psychological stress in virtual urban environments

**DOI:** 10.1007/s10055-025-01300-3

**Published:** 2026-01-17

**Authors:** Reza Aghanejad, Amit Birenboim, Mario Matthys, Sebastien Claramunt, Camille Perchoux

**Affiliations:** 1 Luxembourg Institute of Socio-Economic Research, Esch-Sur-Alzette, Luxembourg; 2 University of Luxembourg, Esch-Sur-Alzette, Luxembourg; 3 Hebrew University of Jerusalem, Jerusalem, Israel; 4 Ghent University, Ghent, Belgium; 5 Vrije Universiteit Brussel, Brussels, Belgium

**Keywords:** Urban Environment, Walking, Virtual Reality, Physiological Stress, Psychological Stress, Biosensor

## Abstract

**Introduction:**

Stress is a growing public health concern, with urban environments strongly influencing stress levels. Experimental approaches simulating human motion (i.e., walking) in virtual urban environments may constitute a promising avenue to assess environmental effects on stress. This study aims to systematically examine the ability to induce and measure psychological and physiological stress responses during walking in immersive virtual urban environments. It includes two sub-experiments: one comparing average stress levels between an urban park and a street, and one assessing momentary stress responses to a siren stimulus.

**Methods:**

Fifty adults residing in Luxembourg experienced virtual walks through a park and a street, in a randomized crossover design, as well as a walk through the street with a siren stimulus. Physiological responses (electrodermal activity (EDA), heart rate (HR), and pupil diameter) were recorded continuously, while self-reported stress and emotional perceptions were assessed after each session via in-VR questionnaires.

**Results:**

Participants reported significantly lower self-reported stress and more positive emotional engagement in the park compared to the street. The standard deviation of HR was higher in the street, possibly indicating higher stress levels, while average pupil diameter was larger in the park, reflecting heightened emotional arousal. Other biomarkers showed no significant differences. Pupil diameter and EDA effectively detected momentary stress after the siren stimulus.

**Conclusions:**

Combining virtual environments with a walking simulator and biosensors provides a novel, effective method to study environmental impacts on stress. Findings contribute to identifying stress-inducing urban environments and developing stress reduction interventions.

**Supplementary Information:**

The online version contains supplementary material available at 10.1007/s10055-025-01300-3.

## Introduction

Stress negatively impacts mental and physical health (Ribeiro Santiago et al. 2020). It is a major contributor to mental disorders, autoimmune, infectious and cardiovascular diseases, and some cancers (Schneiderman et al. 2005; Wirtz and von Känel 2017). Stress further increases the likelihood of adopting unhealthy behaviors such as smoking, alcohol and substance use, physical inactivity, and unhealthy diet (Khaled et al. 2020), which may influence chronic diseases. As stress prevalence has risen over the last decade (Ribeiro Santiago et al. 2020), prevention has become a public health priority.

Exposure to daily life environments plays an important role in shaping population stress levels (Fernandes et al. 2021; Berto 2014). Many studies have provided strong evidence of the positive effect of exposure to nature on stress (Shuda et al. 2020; Berto 2014; Yao et al. 2021; Twohig-Bennett and Jones 2018; Kondo et al. 2018), often explained by the stress reduction theory (SRT) (Ulrich 1983). SRT, utilizing its psycho-evolutionary framework, elucidates how natural environments are anticipated to reduce psychological and physiological stress. The theory posits that the evolution of humans in natural environments over a long time has led to the development of adaptive responses to natural features (e.g., vegetation, water), such that exposure to these elements elicits immediate, unconscious affective responses that enhance psychological, physiological and behavioral stress responses. Ulrich’s original laboratory experiment demonstrated faster and more complete stress recovery in natural compared to urban settings across physiological responses, accompanied by increased positive affect (Ulrich et al. 1991). Subsequent evidence has been synthesized in several reviews. They consistently conclude that exposure to nature reduces both perceived and physiological stress, including evidence from laboratory and field studies (Berto 2014; Shuda et al. 2020; Yao et al. 2021; Kondo et al. 2018). Although less central to stress per se, the Attention Restoration Theory (ART) (Kaplan and Kaplan 1989) offers a complementary cognitive perspective. According to ART, environments that lack certain restorative qualities—such as fascination, a sense of being away, extent (richness and coherence), and compatibility with individuals’ goals—fail to support the recovery of depleted attentional resources. Urban environments, which lack these qualities, are less restorative than natural settings that effortlessly capture attention and support recovery. Moreover, urban environments characterized by continuous environmental demands (e.g., noise, crowding, and visual complexity) impose increasing loads on cognitive resources. These heightened demands can lead to mental fatigue (Kaplan 1995), which in turn increases vulnerability to stress (Kaplan 1995; Ulrich et al. 1991), or—when the demands exceed an individual’s coping capacity—can directly induce stress (Evans and Cohen 1987; Cohen 1978). In contrast to natural environments, evidence on the effects of other urban environmental characteristics on stress is quite limited (Weber and Trojan 2018) and includes mainly urban environmental stressors such as urban density (Baumann and Brooks-Cederqvist 2023; Knö et al. 2017), traffic intensity and noise (Kou et al. 2020; Tao et al. 2020; Yang and Matthews 2010). Considering that 68 percent of the world’s population is projected to live in cities by 2050 (United Nations 2018), and acknowledging the scarce and unequal opportunities for regular contact with nature, there is a need to better understand the stressful or restorative qualities of everyday urban landscapes.

Studies investigating the effect of urban environments on stress use either field experiments (Birenboim et al. 2019a, b; Tyrväinen et al. 2014; Ojala et al. 2019; Olszewska-Guizzo et al. 2020), which offer greater ecological validity, or lab-based experiments (Van den Berg et al. 2014; Helena Nordh et al. 2013; Helena Nordh and Østby 2013; Xinxin Wang et al. 2016; Jiang et al. 2015; Ho and Chiu 2021; Reece et al. 2022; Martínez-Soto et al. 2019), which are considered to have higher internal validity for causal inference (Birenboim et al. 2019a, b). Virtual reality (VR) technology, with its highly realistic and immersive environments, emerges as a potential solution to the long-standing dilemma of balancing experimental control and ecological validity (Birenboim et al. 2021). Immersive virtual environments (IVEs) enable highly controlled, lab-based experiments while mimicking real-world complexity and realism (Birenboim et al. 2021). To date, only a few experimental studies using IVEs have focused on the restorative effect of contemplative/static exposure to natural scenes (e.g., green and blue spaces) (Anderson et al. 2017; Xiaobo Wang et al. 2019; Gao et al. 2019; Tabrizian et al. 2018; Hedblom et al. 2019; Calogiuri et al. 2018; Hian et al. 2021; Annerstedt et al. 2013). Alternative experimental methods include window/facade views (Elsadek et al. 2020, 2019), photographs (Mahamane et al. 2020), and videos (Reece et al. 2022; Xinxin Wang et al. 2016), and are limited by a two-dimensional sensory experience (Xiaobo Wang et al. 2019) and the typically stationary postures involved (e.g., sitting or standing still). However, exposure to urban environments mostly occurs while individuals are moving through space. This movement may affect individuals' emotional responses through spatial sequence arrangement and shifting scenario sequences (Li et al. 2016). Therefore, considering human movement while studying the effect of environmental exposure on stress is of importance. It ensures that stress responses are measured in realistic, dynamic interaction with environments. IVEs make it possible to implement locomotion devices such as a walking simulator in lab-based experiments, increasing ecological validity by mimicking individuals’ real-world experience of moving through urban environments. Compared to walking-in-place (Batistatou et al. 2022; Hian et al. 2021), joystick-driven movement (Hian et al. 2021), or teleportation (instantly jumping from point to point using a controller), treadmill-based walking generates greater immersion and a stronger sense of presence (Boletsis and Cedergren 2019). It also helps mitigate the likelihood of cybersickness, which is a form of motion sickness arising from sensory conflicts between visual and vestibular inputs in VR (Reason and Brand 1975).

Previous experimental studies examining the effect of environments on stress mostly relied on either psychological (Honey-Rosés and Zapata 2023; Beute and de Kort 2018; Tao et al. 2020; Lindal and Hartig 2013; Helena Nordh et al. 2013; Helena Nordh and Østby 2013) or physiological (Walker et al. 2016; Jiang et al. 2014; Kitabayashi et al. 2015; Engelniederhammer et al. 2019; Zhang, Danish, et al. 2022; Zhang et al. 2022; Benita and Tunçer 2019; Annerstedt et al. 2013) stress measurements. Psychological measures directly capture individuals’ perceptions and feelings (Corneille and Gawronski 2024) however, they are susceptible to self-report and social desirability biases (Reece et al. 2022; Birenboim et al. 2019a, b). On the other hand, physiological measures offer insights into participants' autonomic responses; though, they are at risk of external interferences (Bi 2025). These interferences can result in mismeasurement, particularly in ambulatory studies (Bigazzi et al. 2021; Bi 2025). Very few studies combined both stress measurement when assessing the relationships between environments and stress, and reported mixed evidence. Notably, Beil and Hanes (2013) highlighted inconsistencies between two measurements, reflecting the complexity of stress responses. This lack of concordance between the two measurements was also reported by Reece et al. (2022). Considering that physiological autonomic responses may differ from conscious self-reported responses (Beil and Hanes 2013), implementing both as complementary measures can ensure a more comprehensive and accurate understanding of the environment-stress relationships (Baumann and Brooks-Cederqvist 2023; Beil and Hanes 2013).

Physiological stress responses are closely tied to the activity of the autonomic nervous system (ANS) (Jansen et al. 1995). Through its two branches—the sympathetic and parasympathetic nervous systems—the ANS regulates bodily functions such as cardiovascular activity, respiration, sweat gland activity, digestion, pupillary responses, and sexual arousal (Kreibig et al. 2007). Accordingly, physiological signals from the ANS derived from different bodily systems can be used to infer an individual’s stress responses (Birenboim et al. 2019a, b). Among them, heart rate (HR) and electrodermal activity (EDA) are most commonly used in stress research (Moser et al. 2023; Yu et al. 2018), in part because they can be recorded reliably and non-invasively in ambulatory settings (Birenboim et al. 2019a, b). The sympathetic activity during a fight-or-flight response in situations perceived as overwhelming increases heart activity to prepare for action (Kreibig et al. 2007), whereas the parasympathetic activity induces a lower HR (Taelman et al. 2009). An elevated HR is associated with stress (Kim et al. 2018; Taelman et al. 2009) and emotions of anger, anxiety, embarrassment, fear, happiness, joy, and surprise (Birenboim et al. 2019a, b). Lower HR, on the other hand, is linked to a state of serenity and emotions such as acute sadness, affection, and contentment (Kreibig 2010). EDA reflects changes in skin conductance driven by sweat gland activity, which is regulated by the sympathetic nervous system. Raw EDA signals are typically divided into two components: (1) the tonic component, or skin conductance level (SCL), reflecting the baseline level of skin conductivity, and (2) the phasic component, or skin conductance responses (SCRs), reflecting phasic increases in conductivity amplitude of skin. High EDA levels are linked to psychological stress and negative emotions, whereas lower levels are associated with calm or restorative states (Healey and Picard 2005; Kreibig 2010).

IVEs enhance the quality of both psychological and physiological measurements. They allow questions to be asked within immersive environments while participants are still wearing the headset. This ensures that participants respond in the moment, rather than recalling their feelings afterward, thus limiting recall biases (Birenboim et al. 2021). Moreover, IVEs make it possible to implement advanced behavioral and physiological measurements through eye-tracking technology. They specifically enable measuring pupil diameter as an indicator of emotional arousal (i.e., low or high) (Skaramagkas et al. 2023; Bradley et al. 2008; Steinhauer et al. 2022; Partala and Surakka 2003; Raiturkar et al. 2016) and cognitive load (Skaramagkas et al. 2023; Mathôt 2018; Steinhauer et al. 2022; Kret and Sjak-Shie 2019). Pupil diameter provides a valuable real-time index of how individuals respond to emotional or cognitive demands in their environment. An increase in pupil diameter, after accounting for luminance effects, indicates heightened sympathetic arousal or increased cognitive activity. This response captures both emotional arousal (Bradley et al. 2008; Raiturkar et al. 2016) and mental workload (Mathôt 2018; Steinhauer et al. 2022; Eckert et al. 2022; Skaramagkas et al. 2023), thus offering a sensitive measure of engagement with environmental stimuli. In addition to this, studies often measure an average stress level within a specific time window, overlooking stress detection at specific moments in space and time. Therefore, the integration of multiple sensors and physiological responses is particularly useful for studies that aim to distinguish between momentary stress responses at specific time points and overall stress levels over longer exposure duration.

The aim of this study is to systematically examine the ability to induce and measure psychological and physiological stress responses during walking in immersive virtual urban environments. The setup combines immersive virtual environments and an omnidirectional treadmill to simulate realistic urban walking experiences and is equipped with an external biosensor measuring HR and EDA, a head-mounted display (HMD) recording pupil diameter, and in-VR questionnaires to capture both physiological and psychological stress responses. To achieve this aim, we conducted an experiment that comprised two complementary sub-experiments. The first sub-experiment compared exposure to two contrasting urban environments—an urban park and an urban street—to test the ability to detect differences in average stress levels, experienced over a longer exposure period, across two environments. The second sub-experiment, independent of the first one, exposed participants to the same urban street, with an acute stress stimulus (a fire truck with a siren sound appearing from behind) to test the ability in detecting momentary physiological stress responses—referred to as the moment of stress (MOS). By leveraging this innovative approach, we aim to enhance understanding of how environmental characteristics influence both average and momentary stress responses, and to contribute to the methodological advancement of stress research in urban studies.

Based on the primary aim of the study and prior empirical evidence, we formulated the following hypotheses for the two sub-experimental conditions:

*Sub-experiment 1 (average stress levels)*:

### H1:

 Participants will report lower perceived stress following exposure to the park compared to the street.

### H2:

Pupil diameter will be smaller in the park compared to the street, reflecting lower sympathetic arousal and cognitive load. The park’s restorative qualities are expected to elicit calm, effortless engagement, whereas the street’s continuous environmental demands (e.g., noise, car traffic) should evoke greater autonomic activation and mental effort, leading to larger pupils.

### H3:

Heart rate responses will differ between environments: the average HR will be lower in the park compared to the street, reflecting reduced physiological stress, while the standard deviation of HR will be higher in the street, indicating greater variability due to stressful characteristics of the street environment.

### H4:

Electrodermal activity will, on average, be lower in the park relative to the street across all derived indexes, including tonic (e.g., SCL) measures and phasic (e.g., SCR) measures, reflecting reduced sympathetic activation in the park. The standard deviation of EDA will be higher in the street, indicating greater variability of stress responses.

*Sub-experiment 2 (moment of stress)*:

### H5:

Exposure to the fire-truck siren sound will elicit a significant increase in pupil diameter, reflecting heightened sympathetic arousal in response to the acute stressor.

### H6:

Exposure to the fire-truck siren sound will elicit a significant increase in EDA, reflecting heightened sympathetic arousal in response to the acute stressor.

## Method

### Participants

This pilot study included 50 participants recruited through convenience sampling in April 2024. Recruitment was conducted via flyers distributed on the university campus and emails sent through the Luxembourg Institute of Socio-Economic Research (LISER) and University of Luxembourg mailing lists. Based on the literature (Elsadek et al. 2020; Baumann and Brooks-Cederqvist 2023), we assumed a medium effect size of Cohen’s d = 0.50 for a within-subject repeated-measures analysis, with a statistical power of 0.90 and a significance level of 0.05, resulting in a required sample size of 36 participants. To account for potential participant loss due to technical or other reasons, we aimed to recruit 50 participants.

Inclusion criteria required participants to be adults, aged 18–65 years, residing in Luxembourg, with normal or corrected-to-normal vision and hearing, no color blindness, self-reported non-pregnancy, and the ability to walk independently without assistive devices. Exclusion criteria included self-reported diagnoses of heart disease, irregular heartbeat, respiratory diseases, mental disorders, or symptomatic systemic sweating, as well as current medical treatment or medication for these conditions. Fluency in either French or English was also required. These criteria were assessed through an online eligibility questionnaire completed by participants prior to the study.

One participant was excluded from the entire sample for not following instructions during the experiment, leaving a final sample of 49 participants. Written informed consent was obtained from all participants following a detailed explanation of the study procedures. Participants were informed of their right to withdraw at any time without consequences, and the confidentiality of their data was ensured. The study protocol was approved by the LISER research ethics committee (LISER REC/2021/024.FRAGMENT/8). Participants were compensated with a €20 gift voucher for their time and participation.

### Materials

**Fig. 1:**
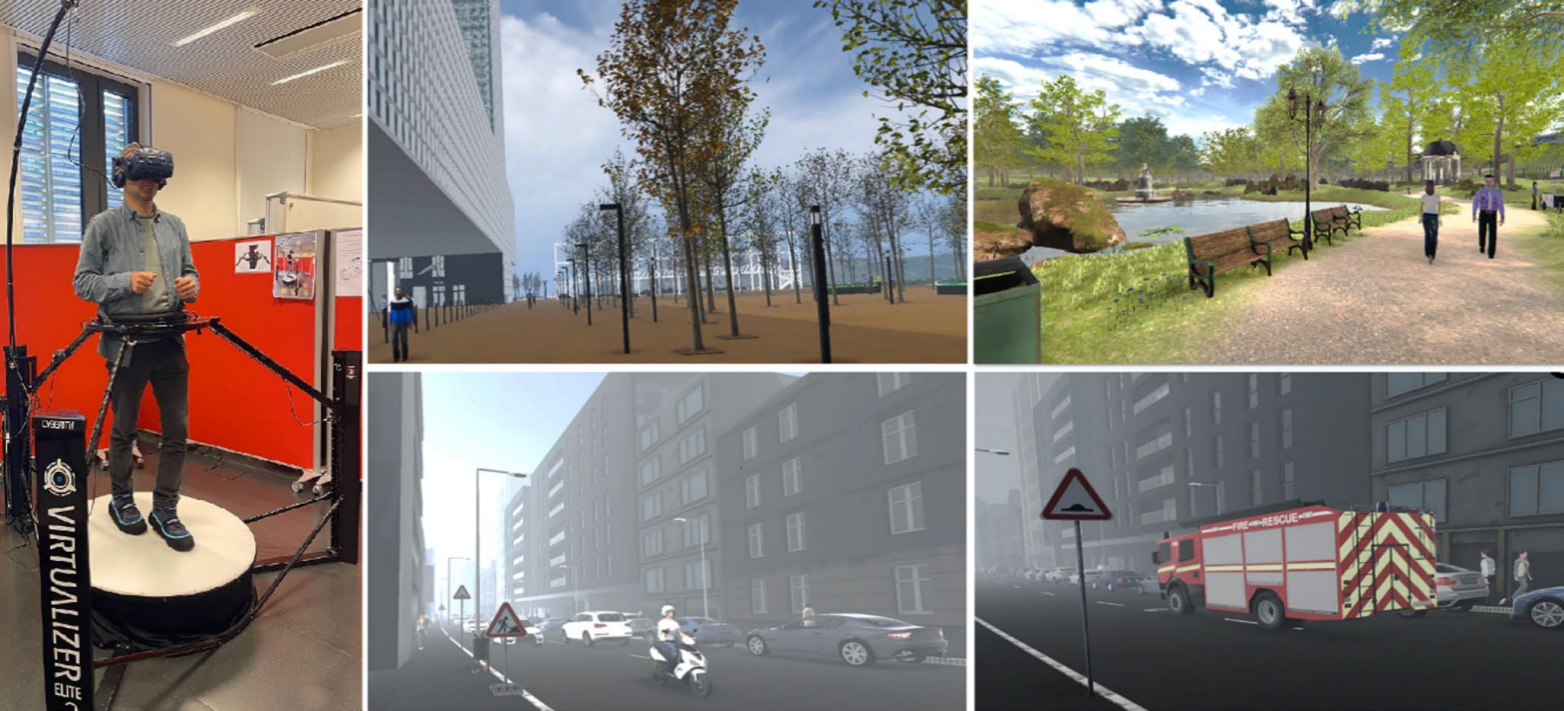
The physical setup of the experiment showing the omnidirectional treadmill and VR headset (left), and screenshots of the four virtual environments used in the study. Top middle: Neutral environment; Top right: Urban park; Bottom middle: Urban street; Bottom right: Urban street with the fire truck included

Three IVEs were developed through a third-party company using Unity®, a game engine software, and presented via the HTC Vive Pro Eye HMD. Participants performed walking tasks on the Virtualizer ELITE 2 omnidirectional treadmill (See Fig. 1), which allowed for a more realistic sense of walking within the IVEs (Birenboim et al. 2019a, b). To meet the study’s goals, two typical yet contrasting urban environments were created—an urban park and an urban street—in addition to a more neutral environment used to train the participant to walk in VR.

*Neutral Environment*: This neutral environment simulated the neighborhood where the VR laboratory is located. It featured low pedestrian and vehicle traffic and minimal ambient noise, providing a controlled setting for participants to acclimate to the VR setup without confounding stimuli (See Fig. 1).

*Urban Park*: The park environment was designed as a typical naturalistic green space within an urban setting. It featured a closed-loop path, grassy areas, a central pond with a fountain, benches, and diverse vegetation (trees, bushes, flowers, etc.). Ambient birdsong enhanced the sensory experience, while virtual people animated with walking movements contributed to the environment's realism. This environment was used to assess average stress responses in a restorative setting.

*Urban Street*: Representing a typical urban street segment with heightened environmental stressors characterized by a densely built area with monotonous high-rise buildings, moderate to high traffic, and urban noise. Features such as overflowing bins, visible litter, “danger” traffic signs, and the absence of green spaces emphasized its stressful nature. This environment was used both to measure and compare average stress levels with the park environment and to detect moments of acute stress during a siren event. For the latter, a fire truck with a loud siren was introduced into the urban street environment during a separate session to simulate a realistic acute stressor.

A figure illustrating screenshots of the park, street, and street with the fire truck during the siren event is presented in Fig. 1.

Physiological responses were measured using two devices. The HMD was used to measure pupil diameter. The Empatica E4 wristband continuously monitored HR, and EDA, providing complementary data for analyzing stress responses. In addition to these physiological measures, psychological responses were measured using questionnaires administered within the VR environment.

### Procedure

**Fig. 2:**
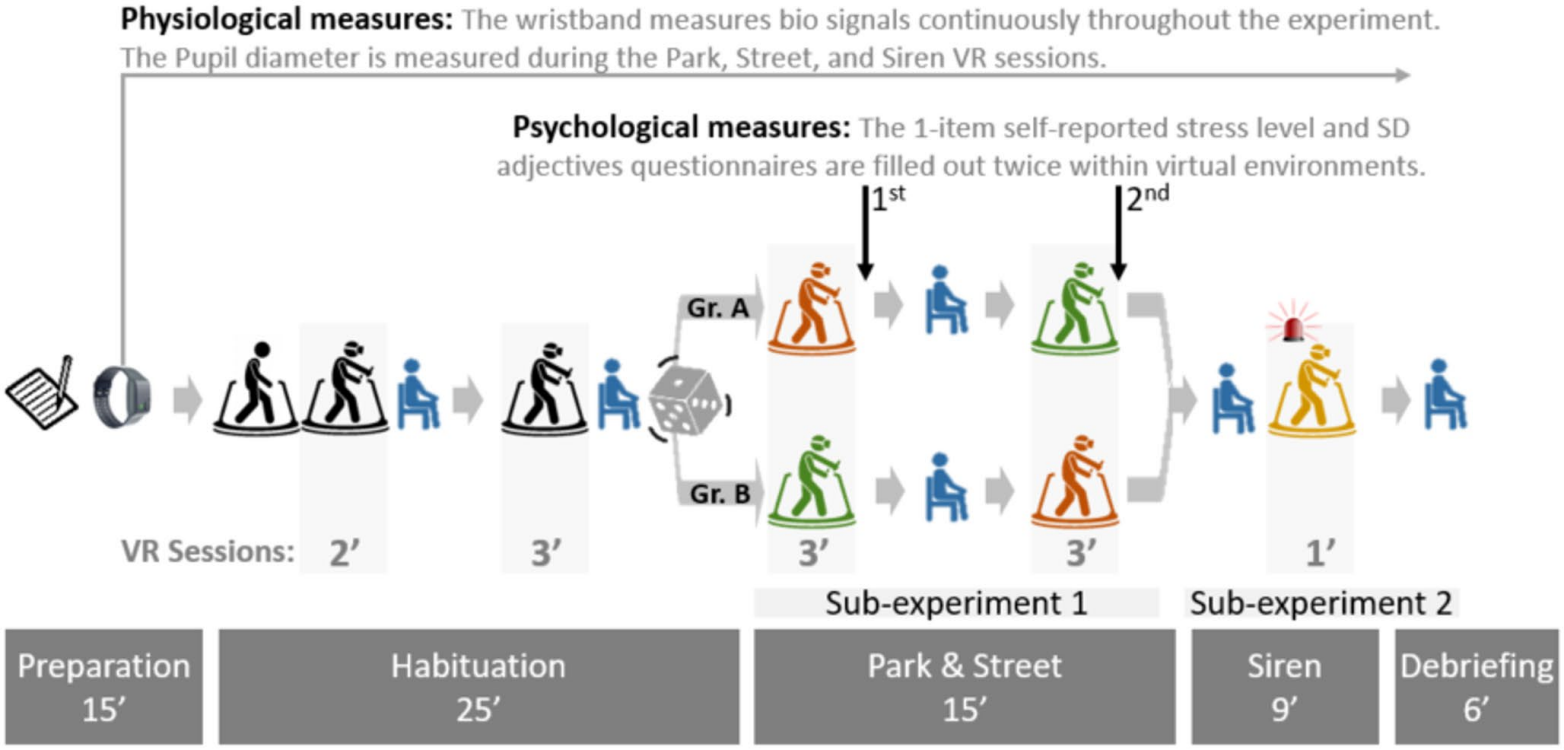
Overview of the experimental design including the two sub-experiments

The experiment was conducted at the LISER VR laboratory in Belval. Recruitment began with the distribution of flyers and emails containing a link to an online eligibility questionnaire. Interested individuals completed the questionnaire, which collected demographic information and assessed inclusion criteria. Eligible participants selected a convenient time slot from a list of available sessions. Upon arrival at the laboratory, participants read an information sheet, signed a consent form, and were fitted with the Empatica E4 wristband. The overall experiment comprised two complementary sub-experiments designed to evaluate different aspects of stress responses. An overview of the experimental design, including the sequence of walking and resting sessions, is shown in Fig. 2. The experiment consisted of three phases: 1) habituation, 2) the first sub-experiment involving exposure to park and street sessions in a random order to assess average stress levels in two contrasting urban environments, and 3) the second sub-experiment involving the siren session to detect moments of stress. The total session lasted approximately 70 min. Five-minute rest periods were incorporated throughout the experiment to allow for physical and physiological recovery, mitigate potential cybersickness, and minimize carryover effects between environments. During the rest periods throughout the experiment, participants also completed additional questionnaires on potential covariates.

*Habituation phase*. The habituation phase was designed to help participants habituate to the treadmill and the VR. It familiarized participants with the setup before the main experiment, minimizing the impact of novelty on participant behavior. This phase consisted of three walking sessions interspersed with rest periods to allow for physical and physiological recovery. Participants began with a 2-min treadmill acclimation walk without the HMD, followed by a 2-min walk while wearing the HMD to adjust to the immersive VR experience. Finally, participants completed a 3-min walk in the Neutral environment. Before starting the main experiment, the eye-tracking calibration provided by the HMD was conducted to ensure accurate pupil diameter measurements. Following calibration, participants were exposed to a black screen and a white screen for 5 s each to collect baseline data for normalizing pupil diameter values based on screen brightness.

*Sub-experiment 1 (Average stress level assessment)*. To assess average stress levels, the experiment employed a within-subjects design in which each participant experienced two conditions: urban park and urban street (without siren sound). A randomized crossover approach was used to control for order effects, with participants randomly assigned to one of two groups. One group started with the park session followed by the street, while the other group experienced the street session first and then the park. Self-reported questionnaires assessing stress level and environmental perception were administered immediately after each VR session while participants remained wearing the HMD.

*Sub-experiment 2 (Moment of stress assessment)*. The second sub-experiment (structured as a within subject design) session was designed to simulate an acute—but realistic—stress event. We implemented the event only in the urban street environment. This choice was made because the purpose was to validate the ability of the setup to capture acute physiological stress responses in an urban scenario, rather than to compare the acute response between environments. To avoid influencing stress responses in the earlier sessions, this session was conducted at the end of the experiment. During this session, a virtual fire truck with a loud siren was programmed to appear from behind the participant at the 20th second of a 1-min walk. The siren lasted 7 s and was calibrated to be noticeable without being overly jarring. The fire truck siren was selected as an acute auditory stimulus designed to induce momentary stress, as sudden alarm sounds have been shown to evoke immediate physiological stress responses such as increased heart rate and cortisol release (Hall et al. 2016). After the session, participants completed the same self-reported questionnaires for stress and environmental perception, administered within the VR environment while wearing the HMD.

*Debriefing and compensation*. At the end of the experiment, participants completed a questionnaire on a PC assessing their cybersickness. This was followed by a brief debriefing session, during which participants were provided with an overview of the study’s objectives and given the opportunity to ask questions. Participants were thanked for their time and effort and were compensated with a €20 voucher.

### Measures

#### Stress markers

Stress markers in this study encompassed both biosignals and self-reported psychological stress measures.

*Psychological Measures*: Self-reported stress was assessed through a single-item question on a 5-point Likert scale: "How stressful did you find the environment?”. Emotional perceptions of the environments were evaluated using the Semantic Differential (SD) questionnaire (Elsadek et al. 2020, 2019), which comprised nine opposite adjective pairs (e.g., Comfortable vs. Uncomfortable) rated on a 5-point Likert scale ranging from -2 to + 2. Both perceived stress and SD responses were assessed in VR, using HMD to answer questions via the direction of the participant’s gaze, immediately after each VR walking session.

*Physiological Measures*: Biosignals were recorded using the Empatica E4 wristband and the HMD. The Empatica E4 wristband continuously recorded EDA and HR throughout the entire experiment, including walking, resting, and habituation phases. Pupil diameter was recorded only during the urban park, urban street, and siren VR walking sessions. Derived stress measures included specific metrics for each biosignal, as detailed in Table 1. The HR was excluded from the MOS analysis because Empatica provides HR values averaged over 10-s spans (Empatica 2021). This temporal smoothing reduces sensitivity to second-by-second changes required for detecting momentary stress responses. To account for brightness effects in the pupil diameter measurement, the luminance levels of the VR screens during the VR walking sessions were recorded via Unity® software. These data were used to normalize pupil diameter values, ensuring that observed changes were not influenced by environmental brightness variations.

**Table 1:** Biosignals and derived stress biomarkers

Biosignals	Definition and mechanism	Stress biomarkers (Indexes)	Expected direction	Purpose of use
EDA	A parameter that describes autonomic changes in the skin’s electrical properties and is divided into two components: - Skin Conductance Level (SCL) - Skin Conductance Responses (SCRs)	EDA average (EDA_avg): Mean of EDA during a session	The higher the EDA_avg, the greater the stress (Birenboim et al. 2021)	Average stress level
		EDA standard deviation (EDA_stdv): Standard deviation of EDA during a session	The higher the EDA_stdv, the greater variability of stress responses (Birenboim et al. 2021)	Average stress level
		EDA amplitude: The second-to-second amplitude of EDA	A significant increase leads to a peak in the EDA values in the presence of a stimulus (Kyriakou et al. 2019)	MOS
		Skin Conductance Level (SCL_avg): Mean of SCL during a session	The higher the SCL, the greater the stress(Cai et al. 2023)	Average stress level
		Number of significant Skin Conductance Responses* (nSCR): Frequency of significant phasic responses during a session	A greater number of significant SCR is associated with higher stress levels (Birenboim et al. 2019a, b)	Average stress level
		Amplitude Sum (AmpSum): Sum of significant SCR amplitudes in μS during a session	Greater SCR amplitude is associated with higher stress levels (Birenboim et al. 2019a, b; Benita and Tunçer 2019; Lee et al. 2024)	Average stress level
		Phasic Maximum (PhasicMax): Peak amplitude of the largest SCR in μS during a session	A greater maximum value of significant SCR is associated with higher stress levels (Lee et al. 2024; Birenboim et al. 2019a, b)	Average stress level
HR	Measures the number of heartbeats per minute, regulated by sympathetic and parasympathetic branches of the ANS. Increases under stress due to heightened sympathetic activity	Average Heart Rate (HR_avg): Mean of heartbeats per minute during a session	Higher HR values are associated with stressful states (Taelman et al. 2009; Kim et al. 2018)	Average stress level
		Heart Rate standard deviation (HR_stdv): Standard deviation of heart beats per minute during a session	The higher the HR_stdv, the greater variability of stress responses (Birenboim et al. 2021)	Average stress level
Pupil Diameter	The measurement of the pupil's size, which changes in response to emotional, cognitive, and environmental stimuli	Average Pupil Diameter (Pupil_avg): Mean pupil diameter during a session	Dilated pupils are associated with the process of emotionally engaging stimuli (Bradley et al. 2008) and cognitive load (Mathôt 2018)	Average stress level
		Pupil Amplitude: Second-by-second changes in pupil diameter	A significant increase leads to a peak in pupil values in the presence of a stimulus	MOS

EDA = electrodermal activity, MOS = moment of stress, SCL = skin conductance level, SCR = skin conductance response, μS = microsiemens, ANS = autonomic nervous system, HR = heart rate. * Significant SCRs are skin conductance responses exceeding a threshold of 0.04 μS (Xiang et al. 2021)

#### Covariates

Measures of presence and perceived exertion were collected after each walking session, as these variables could directly influence participants’ responses. Spatial presence was measured using the Spatial Presence Experience Scale (SPES) (Hartmann et al. 2016), which comprises eight items on a 5-point Likert scale ranging from 1 (= I do not agree at all) to 5 (= I totally agree), with higher scores indicating stronger feelings of presence in the virtual environment. Perceived exertion was assessed with a modified Borg’s Perceived Exertion scale (Borg 1998), which uses a 6–20 rating scale, with higher values indicating greater effort.

Additional questionnaires were administered either before the walking sessions or during rest periods on a PC to gather data on potential covariates and participant characteristics. Before the walking sessions, participants completed questionnaires assessing sociodemographic (age, sex, educational level, main activity, and cultural background), environmental preferences, modes of transportation, experience with VR, well-being (WHO-5) (WHO-5 2024), and 1-item sleep quality. During the rest periods, additional assessments included chronic stress (PSS10) (Cohen et al. 1983), quality of life (WHOQOL-BREF) (WHO 2012), and personality traits (BFI-10) (Rammstedt and John 2007). At the end of the experiment, participants completed a questionnaire about their cybersickness using the CSQ-VR (Kourtesis et al. 2023) and awareness of the study objective.

### Data preprocessing

*Data synchronization*: All IVE events were automatically logged during the experiment, including the start and stop times of each session, the onset of the MOS (fire truck with siren), brightness values, and pupil data. These event markers were exported in log files with precise time stamps. Using these time stamps, we synchronized the IVE log with the biosensor recordings (HR and EDA). In addition, the experimenter manually recorded clock times for events outside of the IVE (e.g., rest sessions, habituation phase). These non-IVE events were subsequently aligned with the IVE and physiological data. This procedure ensured accurate temporal alignment (to the second) between all events and physiological measurements.

*Pupil Diameter*: The preprocessing of pupil diameter data involved several steps to ensure data quality and reliability. First, data from six participants were excluded due to failures in eye-tracking calibration and recording errors, resulting in a final sample of 43 participants. Blink artifacts were addressed by performing linear interpolation (Sege et al. 2020; Wang et al. 2018), where missing pupil values during detected blinks were replaced using the pre- and post-blink values. To minimize noise, a fifth-order Butterworth low-pass filter with a cutoff frequency of 10 Hz was applied (Raiturkar et al. 2016). Additionally, a second low-pass Butterworth filter with a cutoff frequency of 5 Hz was applied before down sampling to 10 Hz to avoid aliasing. The mean pupil diameter of the left and right eyes was computed at each sampling point. To account for the delayed physiological response of the pupil to brightness changes, a lagged brightness value was calculated by shifting the recorded brightness levels by 0.5 s (Eckert et al. 2022). Pupil diameter values were adjusted for brightness effects using a linear regression model (Raiturkar et al. 2016), and the residuals from this model were normalized to ensure non-negative values. This adjustment ensured that pupil diameter measurements accurately reflected physiological responses rather than environmental lighting variations.

*Electrodermal Activity*: Raw EDA data were first inspected for values below 0.01 μS, which indicated a loss of contact between the skin and the electrode and were excluded (Winz et al. 2022). A moving window of 10 s was applied to detect motion artifacts, defined as signal changes exceeding three standard deviations, which were flagged for removal (Xiang et al. 2021). Missing values were then addressed using linear interpolation to ensure continuity in the time series. To reduce high-frequency noise caused by factors such as movement, respiration, or device-related issues, a first-order Butterworth low-pass filter with a cutoff frequency of 5 Hz was applied (Kyriakou et al. 2019). The preprocessed EDA data were subsequently imported into Ledalab (Benedek and Kaernbach 2010), a MATLAB-based tool for skin conductance analysis (Benita and Tunçer 2019; Lee et al. 2024; Cai et al. 2023). Four EDA features (Birenboim et al. 2019a, b; Lee et al. 2024) were abstracted using Ledalab’s continuous deconvolution analysis (CDA) with default parametric settings (Winz et al. 2022; Huang et al. 2020; Cai et al. 2023; Xiang et al. 2021; Lee et al. 2024).

### Data analysis

All analyses were conducted using R version 4.4.1 (Posit team 2024), with statistical significance set at a p-value threshold of < 0.05.

*Analysis of Average Stress Levels*: For each participant, the mean of all stress biomarkers was calculated over the three-minute exposure to each environment (park and urban street) and combined with psychological stress measures to create a condition-level data frame. For EDA and HR, the standard deviation of values during each environment was added as well. Each stress marker was analyzed separately. The normality of the data was assessed using the Shapiro–Wilk test. Based on the results, either paired sample t-tests or Wilcoxon signed-rank tests were applied to compare stress levels between the two environments. A one-sided test was used for all comparisons, reflecting the hypothesis that participants would exhibit higher average stress levels in the urban street environment compared to the park. Cohen’s d_z_ and the rank-biserial correlation (r_rb_) were calculated as measures of effect size for parametric and non-parametric pairwise tests, respectively. To account for potential confounders, linear mixed-effects models (LMMs) were employed using the lme4 package in R. These models included environment (park vs. street) as a fixed effect, covariates and their interaction terms when significant at p-value < 0.05, and participant ID as a random effect to account for repeated measures within individuals. Additionally, Kendall's rank correlations were calculated to assess the concordance between all stress markers within each condition separately.

*Analysis of Moments of Stress (MOS) Related to the Siren Onset*: Temporal changes in pupil diameter and EDA were analyzed using an LMM, with the Time as a quadratic predictor to capture potential non-linear trends and participant ID as a random effect to account for repeated measures within individuals. Based on visual inspection of the time-series plot, three key time points were identified: T0 (siren onset), T1 (peak), and T2 (first local minimum after peak). Data points between T0 and T2 were included in the analysis. Additionally, to determine whether physiological responses at T1 differed significantly from T0, post-hoc pairwise comparisons were conducted.

## Results

### Descriptive statistics

Table 2 summarizes participant characteristics (N = 50). The participants included 25 women and 25 men; their ages ranged from 18 to 57 years, with a mean age of 31.2 years (SD = 8.8). The majority were students (66%), and they had either rarely or never experienced VR. Cybersickness was assessed at the end of the experiment using the CSQ-VR, an eight-item questionnaire on a 7-point Likert scale ranging from 1 (= Absent feeling) to 7 (= Extreme feeling) (Kourtesis et al. 2023). Participants reported a mean score of 1.80 (SD = 0.87), corresponding to absent to very mild symptoms on average. This likely reflects the combined effects of treadmill-based locomotion, habituation, and rest sessions, even among individuals with limited prior VR experience. Due to hardware problems (i.e., calibration and recording issues), E4 and pupil data were only available for 48 and 43 participants, respectively.

**Table 2:** Participants' characteristics (N = 50)

Variables	Mean (SD) / n (%)	Variables	Mean (SD) / n (%)
*Sociodemographic Characteristics*		Cultural Background, n (%)	
Age, mean (SD)	31.2 (8.8)	European	29 (58%)
Female, n (%)	25 (50%)	Non-European	21 (42%)
Education Level, n (%)		**Virtual Reality Experience, n (%)**	
Secondary education	7 (14%)	Sometimes/Very often	7 (14%)
Undergraduate	14 (28%)	Rarely	22 (44%)
Graduate	21 (42%)	Never	21 (42%)
PhD	8 (16%)	**Current Living Environment, n (%)**	
Main Activity, n (%)		Downtown or city center	18 (36%)
Student	14 (28%)	Suburbs	12 (24%)
Student and employed	19 (38%)	Small town or village	20 (40%)
Employed	13 (26%)	**General Health**	
Unemployed	4 (8%)	Chronic Stress, mean (SD)	16.7 (6.3)

### Average stress level comparison: urban park vs. urban street

Table 3 shows participants’ psychological and physiological responses to the park and street environments, testing Hypotheses 1 to 4.

**Table 3:** Comparison of all psychological and physiological responses between two environments

	Park	Street	Test (Paired, 1tail)		Effect size
	Mean(SD)		Test Statistic	p-Value	(95% CI)
*Psychological*					
Self-reported stress level					
I found the environment stressful	0.27 (0.73)	2.29 (1.14)	Z = 5.655	< 0.001	0.95 (0.90,0.97)
Semantic Differential Ratings					
Comfortable	1.63 (0.67)	− 1.02 (1.05)	Z = − 5.796	< 0.001	− 0.97 (− 0.99,− 0.95)
Relaxing	1.39 (1.04)	− 0.78 (1.07)	Z = − 5.294	< 0.001	− 0.89 (− 0.94,− 0.81)
Likable	1.73 (0.49)	− 1 (1.06)	Z = − 5.974	< 0.001	− 0.99 (− 0.99,− 0.98)
Cheerful	1.35 (0.83)	− 1.24 (0.8)	Z = − 5.98	< 0.001	− 0.99 (− 0.99,− 0.98)
Beautiful	1.73 (0.57)	− 1.39 (0.81)	Z = − 6.093	< 0.001	− 1.00 (− 1.00,− 1.00)
Colorful	1.63 (0.64)	− 1.41 (0.96)	Z = − 5.905	< 0.001	− 1.00 (− 1.00,− 0.99)
Attractive	1.43 (1.1)	− 1.31 (0.89)	Z = − 5.872	< 0.001	− 0.99 (− 0.99,− 0.97)
Safe	1.71 (0.71)	− 0.53 (1.08)	Z = − 5.791	< 0.001	− 0.98 (− 0.99,− 0.96)
Pleasing	1.73 (0.6)	− 1.22 (1.01)	Z = − 5.968	< 0.001	− 1.00 (− 1.00,− 1.00)
*Physiological*					
Electrodermal Activity					
EDA average	2.06 (2.94)	2.24 (3.1)	Z = − 0.051	0.522	− 0.01 (− 0.32,0.31)
EDA standard deviation	0.29 (0.33)	0.34 (0.41)	Z = 0.492	0.313	0.08 (− 0.24,0.39)
SCL average	1.92 (2.8)	2.05 (2.93)	Z = − 0.113	0.547	− 0.02 (− 0.33,0.3)
nSCR	37.67 (45.1)	41.79 (48.2)	Z = 1.103	0.137	0.14 (− 0.19,0.43)
AmpSum (µS)	8.9 (13.56)	12.2 (24.65)	Z = 1.253	0.107	0.20 (− 0.13,0.48)
PhasicMax (µS)	0.5 (0.59)	0.53 (0.68)	Z = 0.265	0.398	0.04 (− 0.28,0.35)
Heart Rate					
HR average	83.51 (12.88)	85.25 (12.59)	t = 0.81	0.211	0.12 (− 0.17,0.4)
HR standard deviation	5.72 (3.47)	8.14 (3.93)	t = 3.546	< 0.001	0.50 (0.20,0.80)
Pupil Diameter					
Pupil average	1.51 (0.41)	0.83 (0.33)	t = − 13.808	< 0.001	− 2.07 (− 2.60,− 1.54)

SCL = skin conductance level, nSCR = number of significant skin conductance responses, AmpSum = sum of significant SCR amplitudes, Phasicmax = peak amplitude of the largest SCR, µS = microsiemens

*Psychological Responses*: Wilcoxon signed-rank tests revealed that participants reported significantly lower perceived stress levels in the park (M = 0.27, SD = 0.73) compared to the street (M = 2.29, SD = 1.14, Z = 5.655, *p* < 0.001, n = 49, r_rb_ = 0.95, 95% CI [0.90, 0.97]). Additionally, ratings from the Semantic Differential Questionnaire consistently indicated more positive perceptions of the park across all nine adjective pairs. Participants evaluated the park as significantly more comfortable, relaxing, likable, cheerful, beautiful, colorful, attractive, safe, and pleasing compared to the street.

*Physiological Responses*: As indicated in Table 3, the t-test revealed that participants had a significantly higher standard deviation of HR in the street (M = 8.14, SD = 3.93) compared to the park (M = 5.72, SD = 3.47, t = 3.546, *p* < 0.001, n = 48, d_z_ = 0.50, 95% CI [0.20, 0.80]). The mean of pupil diameter showed significant differences between environments as well, as participants had lower pupil diameter in the street (M = 0.83, SD = 0.33) compared to the park (M = 1.51, SD = 0.41, t = −13.808, *p* < 0.001, n = 43, d_z_ = −2.07, 95% CI [−2.60, −1.54]). Other stress biomarkers (HR average and all EDA-derived) did not show any significant differences between the park and the street.

The potential confounding effects of exposure order, as well as covariates such as SPES, walking exertion, and sociodemographic, were analyzed. SPES scores were above the scale midpoint in both environments (street: M = 3.40, SD = 0.57; park: M = 3.56, SD = 0.64). Walking exertion ratings were comparable across environments (street: M = 12.19, SD = 1.63; park: M = 11.94, SD = 1.69), corresponding to moderate exertion on average. The results of the LMMs revealed that none of these variables confounded the relationship between urban environments and stress. Kendall's rank correlation was computed to evaluate the consistency between stress markers for each environment separately (see Supplementary file A). Self-reported stress and SD questions negatively correlated in both environments, supporting the expected inverse relationship. The strong correlations were also found between EDA-derived biomarkers in both environments. Only in the street environment did average HR weakly correlate with the standard deviation of HR and all EDA-derived biomarkers. However, no significant correlations were observed between self-reported stress and biomarkers.

### Moments of stress assessment: siren sound in an urban street

**Fig. 3:**
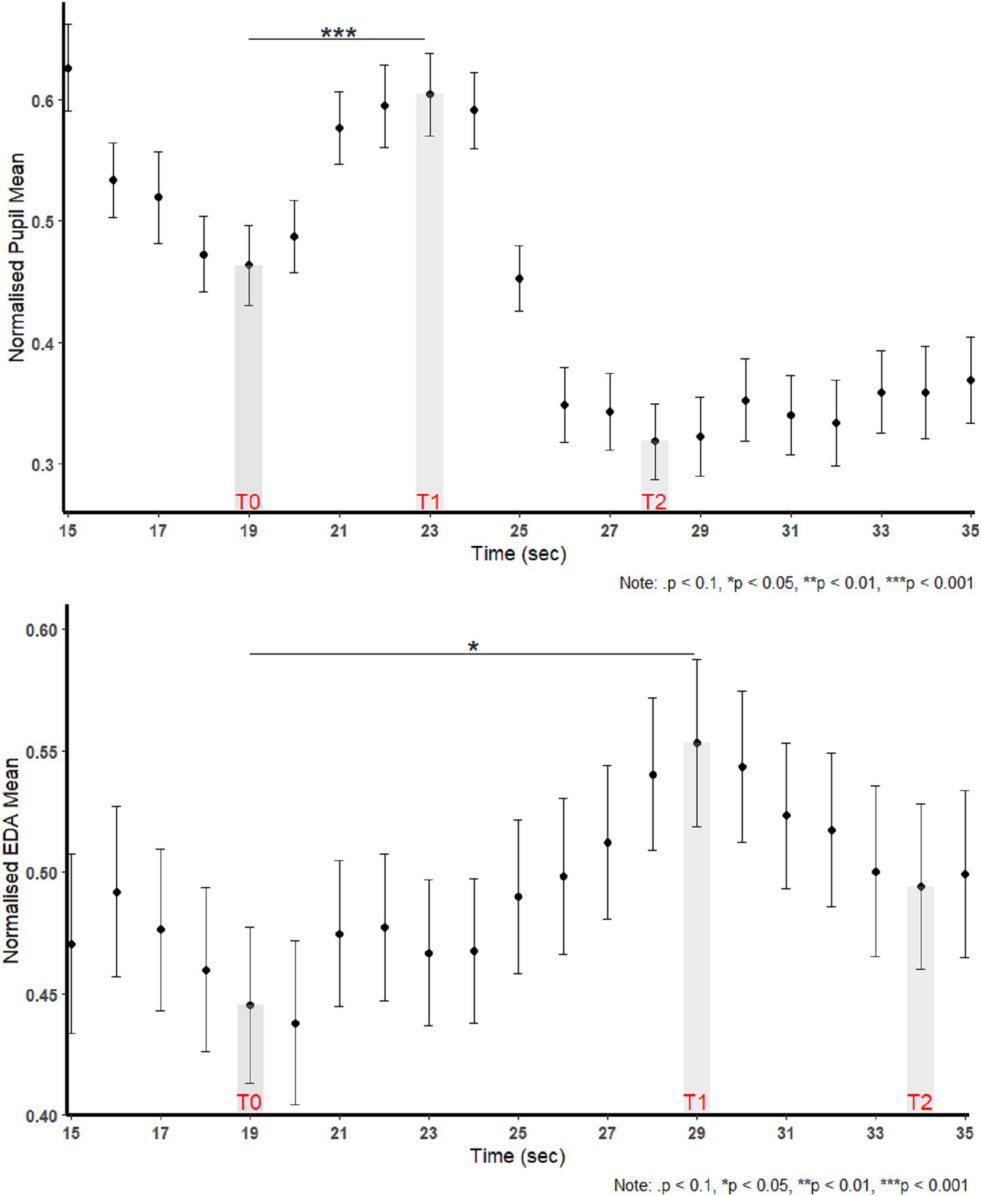
Time-series plots of changes in normalized pupil diameter (top) and EDA (bottom) during walking, showing a 20-s segment of the 1-min walking session. T0 denotes the first onset of the fire truck siren, T1 the post-event peak, and T2 a subsequent decline.

This session examined the effect of a fire truck siren sound, acting as an acute stressor, on participants' physiological (i.e., pupil diameter and EDA) responses, testing H5 and H6. We expected to observe a clear peak in both pupil diameter and EDA following the siren onset. Figure 3 shows the second-by-second changes in mean (a) pupil diameter and (b) EDA before and after the stressor. Both measures exhibited an initial increase following the siren onset (T0 = 19), with pupil dilation responding immediately, whereas EDA showed approximately four to five-second delay, reaching the peak (T1), and a subsequent decline to T2.

*Pupil Diameter Response*: The linear mixed-effects model (LMM) revealed a significant non-linear effect of time on pupil diameter (Time^2^: *p* < 0.001). The results indicate an inverse-U curvilinear pattern of change in response to the siren. As shown in Fig. 3a, pupil diameter increased following the siren onset (T0 = 19 s), reaching a peak at T1 = 23 s, before declining. Pairwise comparisons confirmed a significant increase in pupil diameter from T0 to T1 (estimate = −0.141, SE = 0.039, *p* < 0.001). The result supports the hypothesis that the siren elicited a dilation response following the stressor.

*EDA Response*: Similarly, the EDA response followed an inverse-U curvilinear trend, as indicated by the significant quadratic effect in the LMM analysis (Time^2^: *p* = 0.008). As illustrated in Fig. 3b, EDA showed a delayed increase after T0. The EDA data revealed a peak response approximately 10 s after the onset of the stressor, with an average latency of 3 s and a rise time of 7 s. Post-hoc comparisons revealed a significant increase in EDA from T0 to T1 (estimate = −0.108, SE = 0.0428, *p* = 0.015). It indicates a delayed but measurable autonomic response to the siren stressor.

## Discussion

This study evaluated the feasibility of using a novel experimental setup combining IVEs and an omnidirectional treadmill to simulate realistic walking experiences in urban environments, with the aim of examining how such exposure affects stress. Regarding average stress levels, participants reported significantly lower perceived stress and greater emotional engagement in the park compared to the street. The standard deviation of heart rate was significantly higher in the street, indicating higher variability in HR potentially due to stressful characteristics of the street. In contrast, average pupil diameter was significantly larger in the park, contrary to our hypothesis (H4). This unexpected result might reflect emotional engagement rather than stress, given the visually rich but pleasant context of the park environment. Other stress biomarkers, including all EDA-derived and average HR, showed no significant differences between environments, although the observed trends were in the expected direction. These findings suggest partial consistency between physiological and psychological stress responses. Furthermore, pupil diameter and EDA proved effective in detecting acute stress responses during the fire truck siren event, highlighting their sensitivity as markers of the moments of stress.

Beyond stress outcomes, we assessed key factors related to immersion and cybersickness. Participants generally felt immersed in the IVEs, as the presence ratings were above the scale midpoint in both environments. The realistic design of the virtual environments likely played an important role in enhancing the level of presence. This, together with the use of treadmill-based locomotion, habituation, and rest sessions, also contributed to the very low levels of cybersickness reported. Overall, these results indicate that participants were engaged and physically comfortable during the experiment, supporting the validity of the whole setup for measuring stress responses. Importantly, by integrating multiple biosensors, real-time in-VR data collection, and treadmill-based locomotion within realistic urban environments, this study demonstrates a novel methodological framework for inducing and capturing both prolonged and acute stress responses with high ecological and experimental validity.

### Average stress level comparison: urban park vs. urban street

The positive effects of exposure to natural environments compared to built environments are well documented. Numerous studies highlight the restorative qualities of green spaces in reducing stress and promoting emotional well-being (Weber and Trojan 2018; Yao et al. 2021; Berto 2014; Kondo et al. 2018; Kanning et al. 2022; Hian et al. 2021). According to theories such as SRT (Ulrich et al. 1991) and ART (Kaplan 1995), natural environments provide a capacity to reduce stress and promote emotional engagement. Consistent with these frameworks, this study observed significantly lower perceived stress and greater emotional arousal in the park compared to the street environment, with emotional arousal evidenced by both psychological and physiological responses. These findings validate our experimental setup combining IVEs and walking simulators for assessing average stress levels. It provides a reliable tool for future research in urban environmental stress studies.

We observed significantly different self-reported stress levels between the two environments. Participants found the street environment significantly more stressful than the urban park, thereby validating our first hypothesis (H1). This finding aligns with the study of Beil & Hanes (2013), in which participants reported lower self-reported stress levels in a very natural environment relative to a mostly built setting. The positive effect of exposure to an urban park on self-reported stress was also supported in a meta-analysis conducted by Yao et al. (2021).

Results of the semantic differential questionnaire showed that the urban park scored higher on the emotion “like”, and participants felt more “comfortable”, “relaxed”, “cheerful”, and “safe”. These findings are consistent with Elsadek et al. (2020), in which adult participants reported similar feelings after viewing an urban park through a window compared to an urban built space.

Overall, with regard to self-reported stress, our findings are in agreement with previous studies investigating the restorative effects of exposure to natural scenes, regardless of experimental methods (e.g., real-environment exposure Beil and Hanes 2013; Mennis et al. 2018), window/façade views (Elsadek et al. 2020, 2019), photograph and video (Mahamane et al. 2020; Lindal and Hartig 2015; Xinxin Wang et al. 2016), VR (Huang et al. 2020; Tabrizian et al. 2018; Hedblom et al. 2019; Batistatou et al. 2022; Hian et al. 2021)) or participants posture (i.e., sitting or standing still Elsadek et al. 2019; Huang et al. 2020; Hedblom et al. 2019), walking in place in VR (Batistatou et al. 2022; Hian et al. 2021), and walking in real environment (Brooks et al. 2017; Ojala et al. 2019)). This consistency across various approaches underscores the ability of self-reported measures to capture emotional responses, highlighting participants’ sensitivity to differences in environmental characteristics such as perceived safety, aesthetic appeal, and restorative qualities (Corneille and Gawronski 2024). Self-reported measures account for the emotional experiences of participants, which reinforces their importance in evaluating psychological stress responses in urban studies.

We observed significantly different levels of pupil diameter between the park and the street. Participants exhibited a larger average pupil diameter in the park compared to the street environment, contrary to our hypothesis (H2), which anticipated smaller pupil diameter in the park as a restorative environment. This unexpected finding warrants careful interpretation within the study context. Pupil diameter is a well-established indicator of emotional arousal, mediated by sympathetic activation of the autonomic nervous system (Bradley et al. 2008; Steinhauer et al. 2022; Skaramagkas et al. 2023; Partala and Surakka 2003). Accordingly, a larger pupil diameter would typically be expected in the street, under conditions of heightened stress or cognitive load. However, the absence of larger pupil diameter in the street suggests that this environment did not elicit strong enough stress-related emotional arousal. This interpretation is consistent with the non-significant differences observed in EDA indexes and average heart rate. Recent pupillometry research shows that, while pupil dilation reliably reflects the intensity of emotional arousal, it does not inherently distinguish between positive and negative valence (Skaramagkas et al. 2023; Pei et al. 2022; Bradley et al. 2008; Steinhauer et al. 2022). This limitation underscores the importance of incorporating self-reported measures, such as the SD questions in our study, which are essential for identifying valence direction (Pei et al. 2022; Partala and Surakka 2003). In this study, the SD results consistently indicated more positive emotional engagement in the park, suggesting that the larger pupil diameter reflects positive arousal rather than heightened stress. This interpretation aligns with previous findings showing that pupil size increases during exposure to highly arousing images (Bradley et al. 2008) or auditory stimuli (Partala and Surakka 2003), regardless of whether the stimuli are positively or negatively valenced. Regarding the effect of environmental exposure on pupil size, Martínez-Soto et al. (2019) similarly found that pupil size was larger when participants viewed the images of nature scenes or urban environments with nature, compared to urban built environments without nature. In related studies, these natural images were also rated higher in restorative potential as well as in arousal and pleasure. However, Martínez-Soto et al. (2019) noted their finding contrasted with the Nordh et al. (2010) study, where participants viewing images of small urban green spaces (pocket parks) showed smaller pupil sizes in environments perceived as more restorative, indicating lower physiological arousal. This reported discrepancy might be reconciled by interpreting both sets of findings through the lens of “emotional arousal”. In other words, it is plausible that “restorative” experiences can be either relatively calming (lower arousal, smaller pupils) or positively stimulating (higher arousal, larger pupils), depending on the exact features of the environment and the participants’ perception. Ultimately, both studies align with our own results, indicating that pupil size reflects emotional arousal rather than inherently distinguishing between calming and stimulating forms of restoration.

We observed that the standard deviation of HR measurements was significantly higher in the street than in the park. This supports only the second part of H3, which predicted greater variability in HR in the more stressful street environment. However, no significant differences were found in average HR or any EDA-derived biomarkers between the two environments (H4 not confirmed). However, despite the lack of statistical significance for those other measures, the trend of higher values in the street compared to the park suggests a potential link to increased stress in the street environment. Although HR and EDA are well-documented stress biomarkers (Kondo et al. 2018; Taelman et al. 2009; Healey and Picard 2005; Bolpagni et al. 2024; Hian et al. 2021), the small or non-significant differences observed in our study may be explained by some of the experimental design and technical-methodological considerations.

While we observed significant differences in positive arousal between the two environments, the expected difference in stress level may have been minimal. There might be some explanations. First, According to Cohen (1978) and R. Kaplan and Kaplan (1989), the environment might induce stress if it heavily taxes attentional capacity. Accordingly, individuals have a finite capacity for attention, and as long as this capacity is not exceeded by tasks or demands, potentially stressful environments, such as a dense urban street in our case, may not elicit stress responses. In this study, participants were exposed to environments while walking on a treadmill, without additional cognitive load. This lack of cognitive demands may have allowed participants to allocate sufficient attentional resources to process the environment with all of its competing stimuli.

Second, studies that report significant differences in stress levels between environments often induce stress initially and measure restorative effects afterward (Huang et al. 2020; Xinxin Wang et al. 2016; Hedblom et al. 2019). In contrast, our study aimed to enhance ecological validity by having participants experience walking in different urban environments without initially inducing stress. This approach mimics real-world environmental exposure where individuals naturally walk through urban environments. However, the absence of initial stress induction in our study may have contributed to the lack of significant differences observed between the park and street environments.

Third, the relatively short duration of exposure (3 min per environment) in our study may have been insufficient to detect significant changes in physiological stress levels. Studies such as Huang et al. (2020) observed no significant differences in participants’ SCL levels during the first 5-min exposure to different environments. Prolonged exposures are necessary to observe significant differences in physiological stress responses between environments.

Furthermore, EDA and HR biosignals are sensitive to both emotional arousal and stress, directly linked to sympathetic nervous system activation. While the street environment was hypothesized to be more stressful, the larger pupil dilation observed in the park suggests heightened positive arousal in that environment. Therefore, it is plausible that these biomarkers changed in both environments but through different mechanisms. The change in the street was likely driven by stress responses, while in the park, it was driven by positive arousal. Consequently, the overall changes appeared similar in both environments, potentially masking underlying differences.

From a technical-methodological perspective, the sensitivity of Empatica E4, as noted by Birenboim et al. (2019a, b), may have played a crucial role. Birenboim et al. suggested that the sensor might not be sensitive enough to detect subtle physiological changes that could indicate stress differences between environments. Moreover, devices relying on EDA and HR data are prone to motion artifacts (Tarvainen et al. 2014; Anusha et al. 2020; Birenboim et al. 2019a, b). These artifacts can distort the accuracy of physiological measurements during activities like walking. In our study, in line with Birenboim et al. (2019a, b), the sensor collected incomplete inter-beat interval data sets during walks. Consequently, heart rate variability (HRV), a well-established indicator of stress (Yao et al. 2021; Kim et al. 2018), could not be assessed in this study. This limitation could be addressed by incorporating advanced sensors that are less prone to motion artifacts. For example, using heart rate measurement devices based on electrical methods, such as electrocardiography (ECG), could improve data accuracy and reliability during walking tasks. Unlike photoplethysmography (PPG)-based sensors, which are highly sensitive to movement, ECG provides stable and precise measurements, making it ideal for dynamic experimental setups.

We observed no significant correlations between self-reported and physiological stress measures. This pattern is consistently documented in previous studies showing that physiological autonomic and consciously self-reported stress responses often diverge (Baumann and Brooks-Cederqvist 2023; Beil and Hanes 2013; Reece et al. 2022). Similarly, physiological indicators such as HR, EDA, and pupil diameter did not show strong intercorrelations. The observed discrepancies may partly reflect the technical factors discussed above, including differences in sensor sensitivity and susceptibility to noise artifacts that affect some sensors more than others (e.g., sweating, hand movement, or physical activity). Moreover, while all three physiological indicators are influenced by sympathetic activation, they may measure related but not identical constructs. Consistent with prior findings, EDA and pupil diameter both increased following exposure to the acute stimulus (Bradley et al. 2008). This pattern supports the interpretation that both measures capture momentary stress-related arousal triggered by sudden, high-intensity stimuli. In contrast, during prolonged exposure to the park and street environments, the relationships among physiological measures were weaker and more variable. A weak but significant correlation between HR and EDA-derived indexes was observed only in the street environment. This suggests partial convergence of sympathetic activation under more demanding environmental conditions. Such weak or inconsistent relationships have also been reported in previous studies (Birenboim et al. 2019a, b). However, in a more relaxed environment with reduced sympathetic activation, no significant correlations among physiological indicators were found, consistent with previous findings particularly under non-stress-induced conditions (Beil and Hanes 2013). The larger pupil size observed in the park was also consistent with prior findings (Martínez-Soto et al. 2019) showing greater pupil dilation in natural settings, likely reflecting visual engagement or positive arousal rather than increased stress. Taken together, these results and previous evidence suggest that the correlation between physiological measures is context dependent, emerging primarily in more stressful or cognitively demanding settings which may explains their weak intercorrelations under conditions of moderate or low stress.

### Moments of stress (siren session)

We investigated the temporal change of EDA and pupil diameter as physiological markers of stress during a siren session. Similar to prior research using physiological responses to detect moments of stress following a stimulus (Christopoulos et al. 2019; Kyriakou et al. 2019), this study observed peaks in both EDA and pupil diameter time-series data seconds after the onset of the fire truck siren sound (H5 and H6 confirmed). Previous study has shown that emergency alarm sounds can evoke acute physiological stress reactions, including increased heart rate and cortisol levels (Hall et al. 2016), supporting the interpretation of this response as a momentary stress response. The time-series data indicated that both biomarkers followed an inverse-U curvilinear pattern following the siren sound. However, they differed in characteristics such as latency and rise time. Pupil diameter showed an almost immediate response, while EDA responses demonstrated a slight delay. The results revealed a significant effect of time on both signals. While a clear peak was observed in time-series responses of both signals following the stressor onset, the characteristics of the peaks were different. EDA exhibited approximately a 5-s latency and a 5-s rising time, with the peak observed 10 s following the siren onset. Both the observed latency and rising time are consistent with previously reported average ranges of 1 to 5 s in the literature (Levinson and Edelberg 1985; Kyriakou et al. 2019). In contrast, pupil diameter responded to the stressor almost immediately, with a rising time of approximately 4 s to reach its peak.

Although our findings revealed notable differences between the temporal dynamics of EDA and pupil diameter, both measures converged as indicators of stress-induced arousal, consistent with previous research linking them to sympathetic nervous system activation (Bradley et al. 2008). These findings contribute to understanding the temporal dynamics of physiological responses to acute stressors. Moreover, they highlight the utility of physiological measurements in assessing stress reactions in controlled environments.

### Strengths and limitations

The main strength of our study is the novel combination of highly realistic and immersive IVEs with the omnidirectional treadmill. This setup enabled participants to experience a realistic form of walking through urban environments as their physiological and psychological stress responses were measured via biosensors and in-VR questionnaires. Moreover, the randomized crossover within-subjects design, employing two different environments with a rest session in between, offers the possibility to mitigate ‘order effects’ or ‘carry-over effects’ and produce more robust results. Furthermore, the multiplicity of physiological measures enables us to derive insights into both average stress levels and moments of stress measurements.

A number of limitations of our study need to be acknowledged. First, the number of participants is relatively small (n = 50). It may have impacted the statistical power, potentially masking significant differences in HR and EDA responses between the two environments. Second, the high proportion of students (66%) could affect the generalizability of the findings to the broader population, though the statistical analyses revealed no significant associations or interactions related to sample composition. Third, the study did not control for the possible confounding effects of physical activity, such as walking pace and number of steps. Although participants were monitored and instructed to maintain a consistent and moderate walking pace, some variability may have remained. Finally, the inability to utilize HRV, a well-established stress biomarker, due to motion artifacts is yet another limitation. The Empatica E4 heart rate output was not suitable for MOS detection because it provides values averaged over 10-s windows, which blur second-by-second reactions. Moreover, deriving HR and HRV from the inter-beat interval (IBI) data was not feasible, as the sensor produced incomplete IBI recordings during walking, likely due to motion artifacts. Future studies involving physical activity should implement appropriate sensors to accurately measure stress while moving.

## Conclusion

In this study, we demonstrated the feasibility of using a novel experimental setup that integrates IVEs, an omnidirectional treadmill, and physiological and psychological stress measurements to investigate stress responses in urban environments. Participants reported significantly lower perceived stress and greater emotional engagement in the park compared to the street. The larger pupil diameter in the park suggests heightened emotional arousal, while other biomarkers (EDA and HR) did not show significant differences. Moreover, pupil diameter and EDA effectively captured acute stress responses during the fire truck siren session, highlighting their sensitivity in detecting moments of stress. These findings contribute to the identification of stress-inducing urban environments and the development of stress reduction interventions. Furthermore, this research validates the experimental setup and advances stress measurement methodologies, contributing valuable insights into the urban environment-stress relationship.

## Supplementary Information

Below is the link to the electronic supplementary material.

Supplementary Material 1

## Data Availability

Data are available from the authors upon reasonable request.
