# Supplementary material for: Stressful urban walks: an experimental design for measuring physiological and psychological stress in virtual urban environments
Source: Virtual Real. 2026 Jan 17;30(1):45. doi: 10.1007/s10055-025-01300-3 (PMC12855307; doi:10.1007/s10055-025-01300-3)
Supplement: Supplementary file 1 — Supplementary Material 1 [file 10055_2025_1300_MOESM1_ESM.docx]

**Supplementary file A**

Kendall's rank correlation between self-reported stress and physiological stress markers for each environment separately.

| **Indexes** | Self-reported Stress | EDA (average) | EDA (standard deviation) | SCL (average) | nSCR | AmpSum (µS) | PhasicMax (µS) | Heart rate (average) | Heart rate (standard deviation) | Pupil diameter (average) |
| --- | --- | --- | --- | --- | --- | --- | --- | --- | --- | --- |
| Self-reported Stress | 1.00 |  |  |  |  |  |  |  |  |  |
| EDA (average) | 0.05 | 1.00 |  |  |  |  |  |  |  |  |
| EDA (standard deviation) | 0.11 | 0.716*** | 1.00 |  |  |  |  |  |  |  |
| SCL (average) | 0.03 | 0.986*** | 0.702 *** | 1.00 |  |  |  |  |  |  |
| nSCR | 0.15 | 0.764*** | 0.749 *** | 0.749*** | 1.00 |  |  |  |  |  |
| AmpSum (µS) | 0.12 | 0.775*** | 0.789 *** | 0.76 *** | 0.933*** | 1.00 |  |  |  |  |
| PhasicMax (µS) | 0.04 | 0.734*** | 0.8 *** | 0.723*** | 0.781*** | 0.811*** | 1.00 |  |  |  |
| Heart rate (average) | 0.03 | 0.03 | 0.08 | 0.02 | 0.06 | 0.03 | 0.03 | 1.00 |  |  |
| Heart rate (standard deviation) | 0.04 | -0.08 | 0.00 | -0.07 | -0.03 | -0.03 | -0.05 | -0.05 | 1.00 |  |
| Pupil diameter (average) | 0.09 | -0.06 | -0.05 | -0.05 | 0.00 | -0.01 | -0.06 | 0.07 | -0.01 | 1.00 |
| Note: EDA = Electrodermal activity, SCL = skin conductance level, nSCR = number of significant skin conductance responses, AmpSum = sum of significant SCR amplitudes, Phasicmax = peak amplitude of the largest SCR, µS = microsiemens | | | | | | | | | | |

**1- Park**

**2- Street**

| **Indexes** | Self-reported Stress | EDA (average) | EDA (standard deviation) | SCL (average) | nSCR | AmpSum (µS) | PhasicMax (µS) | Heart rate (average) | Heart rate (standard deviation) | Pupil diameter (average) |
| --- | --- | --- | --- | --- | --- | --- | --- | --- | --- | --- |
| Self-reported Stress | 1.00 |  |  |  |  |  |  |  |  |  |
| EDA (average) | -0.002 | 1.00 |  |  |  |  |  |  |  |  |
| EDA (standard deviation) | 0 | 0.757*** | 1.00 |  |  |  |  |  |  |  |
| SCL (average) | -0.02 | 0.979*** | 0.739*** | 1.00 |  |  |  |  |  |  |
| nSCR | 0.035 | 0.719*** | 0.714*** | 0.697*** | 1.00 |  |  |  |  |  |
| AmpSum (µS) | 0.038 | 0.764*** | 0.794*** | 0.741*** | 0.901*** | 1.00 |  |  |  |  |
| PhasicMax (µS) | 0.008 | 0.752*** | 0.776*** | 0.73*** | 0.782*** | 0.848*** | 1.00 |  |  |  |
| Heart rate (average) | 0.048 | 0.241* | 0.25* | 0.234* | 0.205 | 0.253* | 0.276** | 1.00 |  |  |
| Heart rate (standard deviation) | -0.169 | 0.082 | 0.129 | 0.078 | 0.119 | 0.142 | 0.107 | 0.213* | 1.00 |  |
| Pupil diameter (average) | -0.04 | 0.076 | 0.123 | 0.076 | 0.076 | 0.102 | 0.106 | 0.136 | 0.123 | 1.00 |
| Note: EDA = Electrodermal activity, SCL = skin conductance level, nSCR = number of significant skin conductance responses, AmpSum = sum of significant SCR amplitudes, Phasicmax = peak amplitude of the largest SCR, µS = microsiemens | | | | | | | | | | |
